# Supplementary material for: Natural Killer Cells in Graves’ Disease: Increased Frequency but Impaired Degranulation Ability Compared to Healthy Controls
Source: Int J Mol Sci. 2025 Jan 24;26(3):977. doi: 10.3390/ijms26030977 (PMC11816991; doi:10.3390/ijms26030977)
Supplement: Supplementary file 1 [file ijms-26-00977-s001.zip › ijms-3418607-supplementary captions.pdf]

Supplementary Figure S1: Gating strategy phenotype. Analysis of $CD56^{+}$ $CD3^{-}$ NK cells, $CD56^{bright}$ NK cells, and $CD56^{dim}$ NK cells is shown for activating and inhibitory surface receptors in a Graves' subject (CD16, CD69, NKG2D, NKG2A, NKp30, and CD161).

Supplementary Figure S2: Gating strategy degranulation. Analysis for $CD56^{+}$ $CD3^{-}$ NK cells, $CD56^{bright}$ NK cells, and $CD56^{dim}$ NK cells is shown for CD107a marker with or without K562 in a Graves' subject.

Supplementary Figure S3: Gating strategy intracellular cytokine. Analysis for $CD56^{+}$ $CD3^{-}$ NK cells, $CD56^{bright}$ NK cells, and $CD56^{dim}$ NK cells is shown for IFNγ and TNFα in a Graves' subject.
