# Supplementary figures and images for: Natural Killer Cells in Graves’ Disease: Increased Frequency but Impaired Degranulation Ability Compared to Healthy Controls
Source: Int J Mol Sci. 2025 Jan 24;26(3):977. doi: 10.3390/ijms26030977 (PMC11816991; doi:10.3390/ijms26030977)

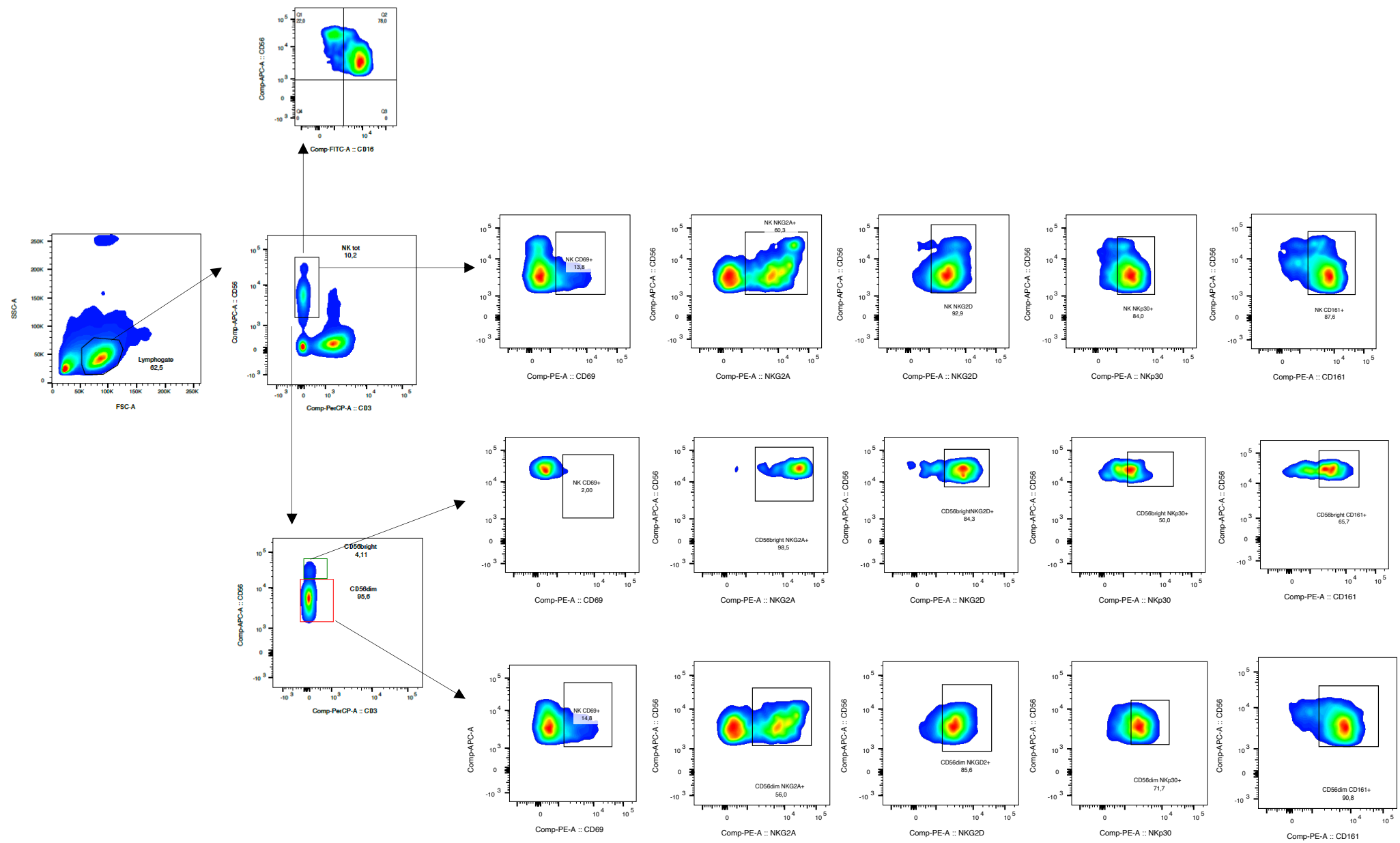

Suppl. Figure 1: Gating strategy phenotype

Supplement: Supplementary file 1 [file ijms-26-00977-s001.zip › FINAL_Suppl. Figure 1- Gating strategy phenotype .pdf]

- K562

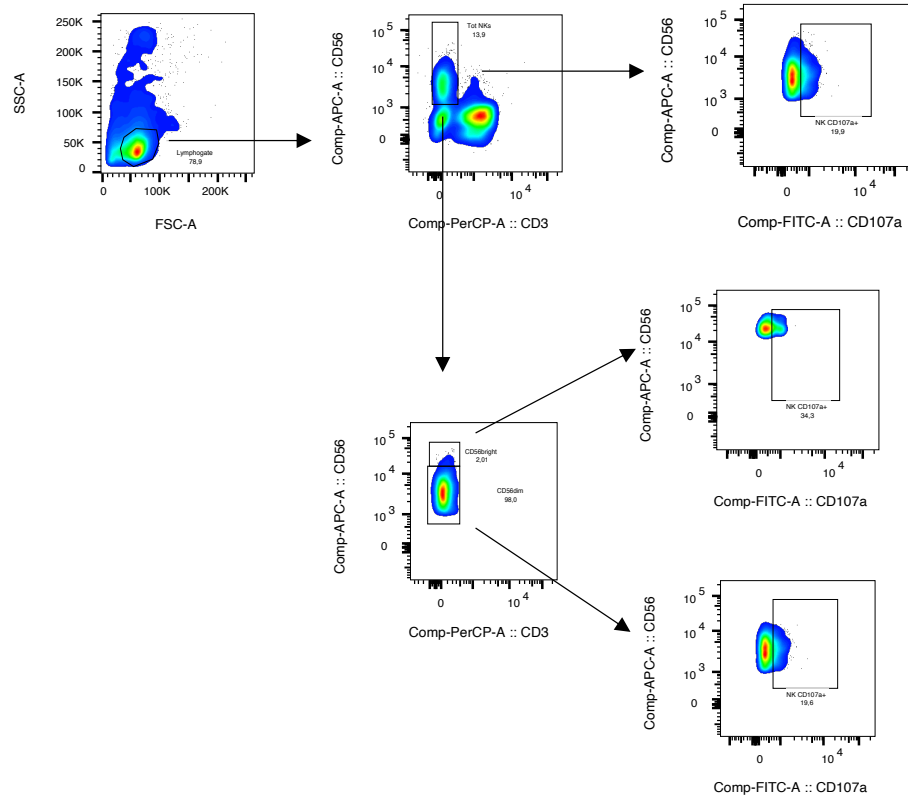

+ K562

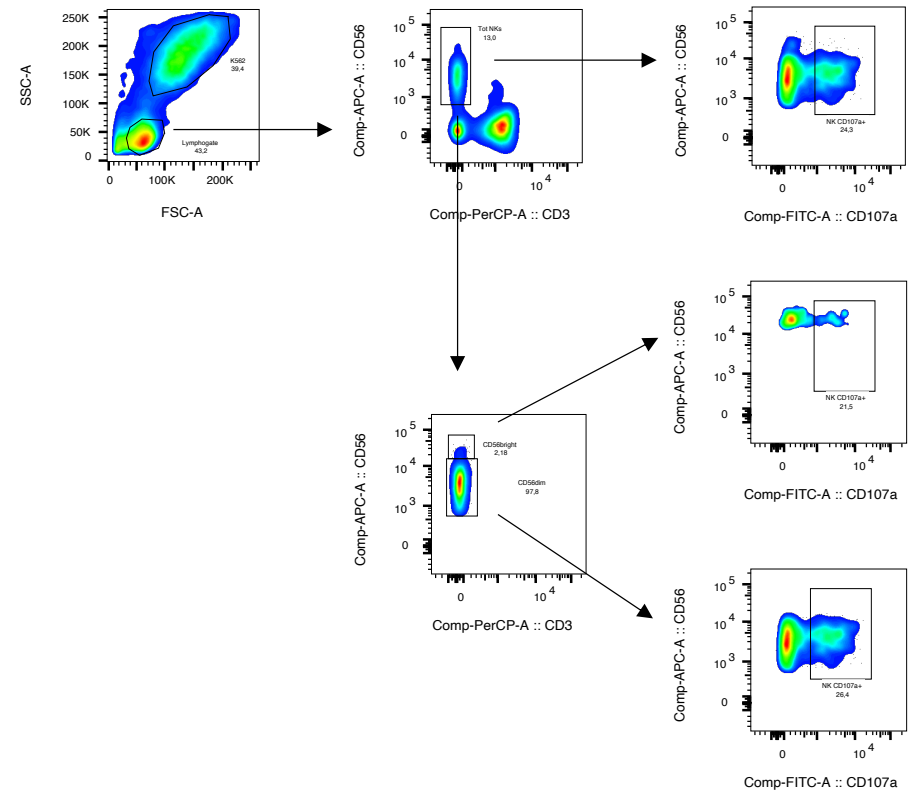

Suppl. Figure 2: Gating strategy degranulation

Supplement: Supplementary file 1 [file ijms-26-00977-s001.zip › FINAL_Suppl. Figure 2- Gating strategy degranulation .pdf]

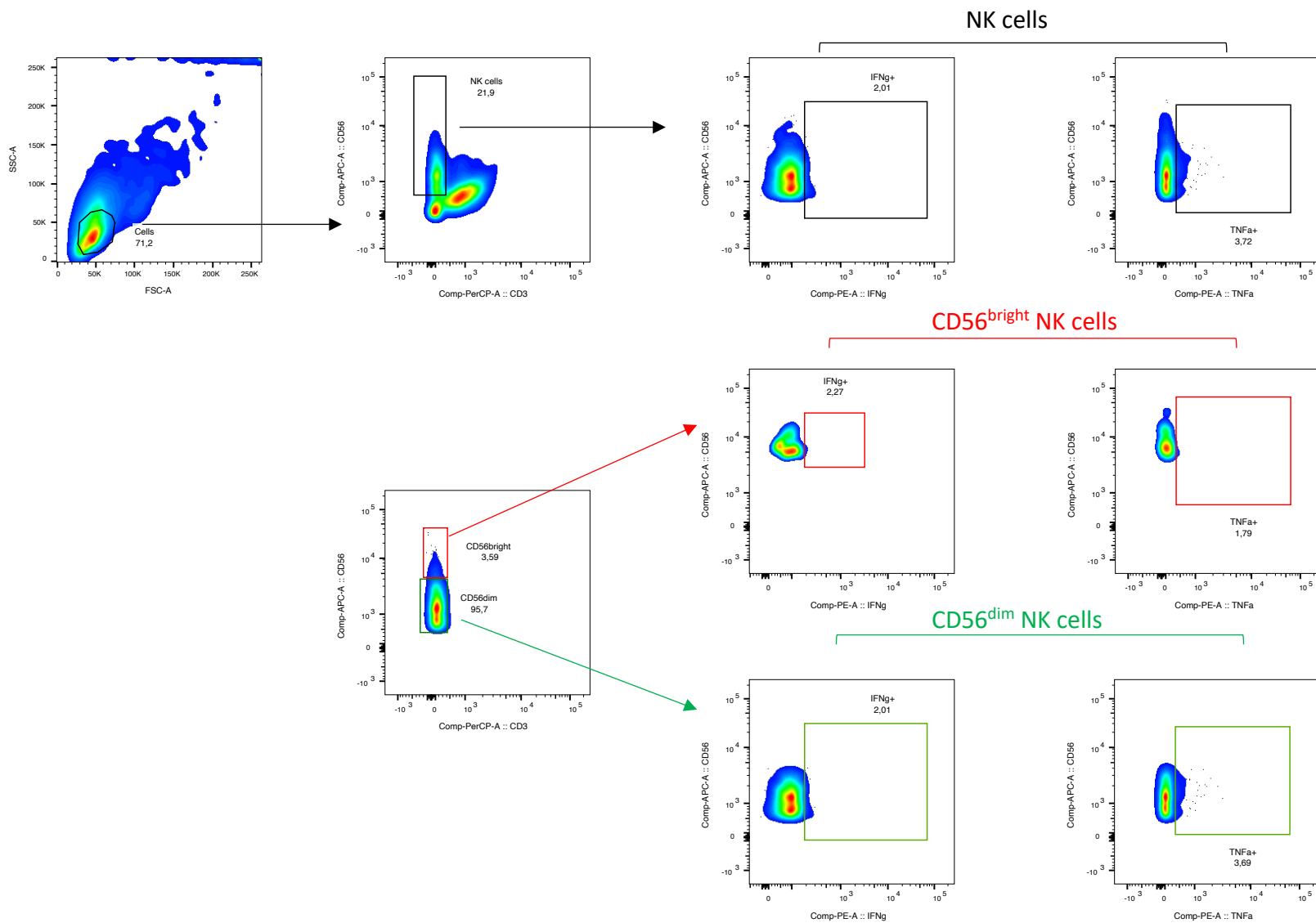

Suppl. Figure 3: Gating strategy ICS

Supplement: Supplementary file 1 [file ijms-26-00977-s001.zip › FINAL_Suppl. Figure 3- Gating strategy ICS .pdf]
